# Small molecule targeting of the p38/Mk2 stress signaling pathways to improve cancer treatment

**DOI:** 10.1186/s12885-023-11319-x

**Published:** 2023-09-23

**Authors:** D. Alimbetov, B. Umbayev, A. Tsoy, D. Begimbetova, T. Davis, D. Kipling, Sh. Askarova

**Affiliations:** 1 Creehey Children’s Cancer Research Institute, UT Health at San Antonio, San Antonio, USA; 2 Center for Life Sciences, National Laboratory Astana, Nazarbayev University, Astana, Kazakhstan; 3 Division of Cancer and Genetics, Cardiff University School of Medicine, Cardiff, UK

**Keywords:** p38/MK2 inhibitors, Small molecules, Cell cycle, Cancer, SCLC

## Abstract

**Purpose:**

Although a long-term goal of cancer therapy always has been the development of agents that selectively destroy cancer cells, more recent trends have been to seek secondary agents that sensitize cancer cells to existing treatment regimens. In this regard, the present study explored the possibility of using small molecule inhibitors of p38MAPK/MK2 stress signaling pathways as potential agents to enhance the sensitivity of cancer cells with abrogated G1 checkpoint to the DNA damaging agent etoposide by specifically targeting the DNA damage-induced G2 cell cycle checkpoint.

**Methods:**

We have applied CCK8 and FACS-based viability assays and cell cycle analysis to investigate the effect of small molecules SB203580 and MK2.III on the sensitivity of small cell lung cancer cells (SCLC) that lack the G1 checkpoint to the DNA damaging agent Etoposide when used in combination. We have also assessed the effectiveness of combination chemotherapy on tumor xenograft suppression with etoposide and MK2.III in immunosuppressed mice. In addition, additional CCK8 cell viability analysis of the MDA-MB-231 breast cancer cell line, and SW620, and SW480 colorectal cancer cell lines was performed.

**Results:**

Results suggest that etoposide produces a profound effect on the cell cycle profile of cells in a manner that is consistent with the degree of cell viability that is seen using the viable cell assay. Results of the co-treatment experiments revealed that the p38/MK2 kinase inhibitors SB203580 and MK2.III both enhanced the DNA-damaging effects of etoposide on NCI-H69 cell viability in vitro. Results revealed that in vivo MK2.III was able to act as a chemosensitizer when used in combination with etoposide making NCI-H69 lung cancer cells sensitive to chemotherapeutic drug by 45% compared to single usage of the drug. We also report that MK2.III sensitizes metastatic cell lines SW-620 and MDA-MB-231 to etoposide but does not increase the sensitivity of non-metastasizing SW-480 colorectal cells to DNA damaging agent in vitro.

**Conclusion:**

Findings reported in this study provide evidence that specific inhibitors of MK2 may indeed improve overall cancer therapy; however, their effectiveness depends on cell types.

**Supplementary Information:**

The online version contains supplementary material available at 10.1186/s12885-023-11319-x.

## Introduction

A key goal in cancer therapy is to kill cancer cells without also killing the normal cells in the patient—a concept known as the therapeutic window. Although a long-term (and still elusive) “Holy Grail” has been the identification of agents that by themselves selectively kill cancer cells, a more recent and nuanced approach has been to seek secondary agents that selectively sensitize cancer cells to existing treatment regimens (e.g. chemotherapy with DNA damaging agents) when used in combination. This approach seeks to exploit knowledge of the consequences of the genetic lesions that are common in cancer cells that lead to altered cell behavior, changes that might, in turn, be exploited for such a ‘chemosensitization’ approach.

One such approach and the focus of this research centers on the cell cycle progression. Targeting cell-signaling pathways and controlling the cell cycle phases and checkpoints is a comparatively new direction for the treatment of cancer which could provide distinctive opportunities and potential for the improvement of cancer treatment outcomes. Cell cycle progression has five known phases: G0 (gap 0), G1, G2, and M. In the G0 phase cells enter a quiescent, resting state. Upon stimulation by growth factors cells enter the G1 phase when the mRNAs and proteins necessary for DNA replication in the S phase are synthesized. In G2, cells are monitored for the integrity and immutability of DNA replication and prepare to start mitosis. In the M phase, the chromosomes and cytoplasm are divided into two daughter cells. There are two important checkpoints at the G1/S and G2/M boundaries [1], at which times the integrity of cellular components and DNA synthesis are monitored, and cell cycle arrest upon DNA damage is regulated by the p53-p21-dependent G1 checkpoint [2], and the Chk1-Cdc25-dependent G2 checkpoint [3, 4]. In turn, tumor cells are tending towards the accumulation of the mutations leading to deterioration in mechanisms of the cell cycle control that results in an altered capacity to respond to DNA damage, and there are a number of cancer therapy strategies focused on the regulatory proteins controlling cell cycle, such as aurora kinase signaling, BRCA1/2, wingless (WNT) signaling, etc. [5, 6].

One of molecules of the great interest is p53. P53 is a cellular tumor antigen that regulates several molecular pathways responsible for cell proliferation and apoptosis [7]. Mutated p53 is typical for many malignant cells, making it an attractive target for anti-cancer therapy. As mentioned, cell cycle arrest upon DNA damage in the G1 checkpoint is p53-p21-dependent [2], and many treatment strategies focus on the reactivation of wild-type functions in the mutated p53 protein [8] or the inactivation of mutated p53. Therefore, a combination therapy using two or more chemotherapeutic drugs or inhibitors to increase the sensitivity of cancer cells to cytotoxic anti-cancer agents could be used to treat p53 mutated cancers, and some animal models have revealed promising outcomes in tumor regression when wild-type p53 was activated in p53 mutated cancers [9]. Yet, such combination chemotherapy is ordinarily focused on p53 mutant cancer cells as p53 impairment causes G1 checkpoint loss [10] therefore leaving cancer cells to rely on the G2 checkpoint for DNA repair and survival. This opens new promises for using inhibitors of G2 checkpoint as chemosensitizers for p53-deficient malignancies [11–13], with several checkpoint kinase inhibitors being currently tested in clinical trials [13].

One of the promising approaches here is targeting ATR/p38MAPK/MK2 pathway. It has been shown that a chromatin-quality checkpoint in late G2 involves ATR/p38MAPK/MK2 [14–16]. The p38MAPK kinase pathway is one of the stress-activated protein kinases involved in apoptotic cell death that acts as key molecules in the apoptotic onset [17, 18] and is associated with several human pathologies such as rheumatoid arthritis [19] and neurodegenerative diseases [20]. The role of p38MAPK kinase in cancer is being widely studied [21] where it was shown to act as an antitumorigenic factor [22] and/or tumor promotor [21]. Downstream of p38MAPK is the mitogen-activated protein kinase activated protein kinase 2 (MAPKAPK-2 or MK2), a kinase involved in inflammatory responses, cell division and differentiation, apoptosis, and cell motility [23]. Furthermore, MK2 is activated after DNA damage [14, 24] resulting in cell cycle arrest and ultimately cellular senescence. These characteristics of p38MAPK and MK2 attract much attention as promising targets for cancer therapy taking into consideration that apoptosis and DNA repair are the main mechanisms associated with cell survival during DNA damage [25, 26].

Work using transgenic mice has recently demonstrated that genetic disruption of the p38MAPK /MK2 pathway specifically sensitizes p53-null mouse cells to DNA-damaging agents [14, 27]. The mechanism of action appears to be that the p53-null cells in the presence of ablation of p38MAPK/MK2 have lost both G1 and G2 DNA damage checkpoint function, and enter mitosis despite the presence of DNA damage, where they die by "mitotic catastrophe". In contrast, the p53 wild-type cells can still arrest in response to DNA damage even in the absence of p38MAPK/MK2 pathway function because the p53-dependent G1 checkpoint remains active. These cells halt in G1 and do not enter into mitotic catastrophe. This finding mirrors the data of our collaborators from Cardiff University [unpub obs] where they have shown that small molecule inhibitors of p38MAPK can preferentially sensitize human fibroblasts with abrogated p53 (RNAi knock-down) to DNA damaging agents, suggesting a similar mechanism could also be functional in cancer cells. In this regard, the present study explored the possibility of using small molecule inhibitors of p38MAPK/MK2 stress signaling pathways as potential agents to enhance the sensitivity of cancer cells with abrogated G1 checkpoint to the DNA damaging agent etoposide by specifically targeting the DNA damage-induced G2 cell cycle checkpoint.

## Materials and methods

### Cell culture and drug treatment

The bulk of the data has been generated with the SCLC cell line NCI-H69, with additional data from the colorectal cancer cell lines SW-480 and SW-620, and breast cancer cell line MDA-231 which also possess mutated p53 gene. The cells were grown in 12 well plates (Scientific Laboratory Supplies Limited, UK) in RPMI 1640 (Gibco, UK) supplemented with 10% FBS (Autogen Bioclear, UK) and 10,000 U/ml penicillin and 10 mg/ml streptomycin (Sigma, UK) at 37 °C under 5% CO_2_ and treated with 10, 20, 50, 100 and 150 µM of DNA damaging agent etoposide (VP-16) (Sigma, UK) in the presence or absence of p38MAPK inhibitor SB203580 (Tocris Chemical Co., UK) or MK2 inhibitor MK2.III (Merck, UK) in concentrations of 0, 0.3125, 0.625, 1.25, 2.5, and 5.0 µM. Etoposide, SB203580 and MK2.III were all dissolved in 0.2% of DMSO before use. Cells treated only with the carrier molecule DMSO (0.2%) served as a control.

### FACS-based cycle analysis and viability assays

Viability analysis was performed by staining with the cell-permeable, stoichiometric DNA stain DRAQ7 (640 or 633 nm excitation and 670 nm emission; Biostatus Limited). After 48-h treatment with DNA damaging agents and/or inhibitors, cells were washed once with RPMI 1640 and resuspended in 1 ml culture media at a concentration of less than 5 × 10^5^/ ml. Cells were stained with 20 µM of DRAQ7 and incubated at 37 °C for 10 min before FACS Calibur cytometer analysis. Fluorescent signals were amplified using linear mode. Cell cycle analysis was performed either by staining DRAQ7 or Propidium Iodide (PI). Cells were stained with 20 µM of DRAQ7 and incubated at 37 °C for 10 min before analysis on a FACSCalibur cytometer. Propidium Iodide (PI, excited with 488 nm laser, collected with 585/52 bandpass filter) was used in the large-scale experiments. Samples were then fixed with 70% EtOH following the viability assay and stored at 4 °C overnight before the experiment. After fixation with 70% EtOH (added drop-wise) for 24 h, samples were centrifuged into the pellet and then washed twice with ice-cold PBS. The supernatant was discarded and pellets were resuspended in 100 µl of RNase A (100 µg/ml) and 400 µl of PI (50 mg/ml in PBS). Analysis of flow cytometry data was performed using FlowJo software. Untreated, unstained cells were used as a negative control to determine the location of viable cells on the dot plots (). Events below a threshold size were interpreted as cellular debris and were removed from the dataset, leaving a whole cell population. The viable cells in this population were then determined via DRAQ7 staining; healthy cells do not stain positive for the stain DRAQ7 as their cellular membrane is still intact, all other cells stain with DRAQ7 and are thus considered nonviable.

### CCK8-based cell viability assays

To assess the effect of MK2.III kinase inhibitor and the etoposide (VP-16) on the viability of different cancer cell lines, cells were seeded in 96-well plates in a concentration of 5000 cells per well. After 12 h, etoposide and 1 μM MK2.III diluted in culture media were added into the wells for 24 h. NCI-H69 cells were exposed to a series of etoposide concentrations from 20 μM to 150 μM. MDA-MB-231, SW 620, and SW 480 cell lines were incubated with 150 μM etoposide. After treatment, 10 μL of the CCK-8 reagent (96992, Sigma) was added to each well for 1 h followed by optical density measurement at 450 nm using hybrid plate reader Synergy H1.

### In vivo experiments

The in vivo set of experiments was performed using the MK2 pathway inhibitor MK2.III based on the outcomes of our previous in vitro studies (higher effectiveness in lower dosages, see Table 2). All procedures related to the animal study were performed according to the protocols approved by the Local Ethics Committee of National Laboratory Astana (Registration number IORG 0006963). Animals were kept in normal vivarium conditions with a scheduled day/night cycle, at a temperature of 22–23 °C, and receiving standard feed and drinking water ad libitum. All mice had the acclimation period of 7 days before use in in vivo experiments. Simple randomization was performed by using a Microsoft Excel spreadsheet. Mice used in the experiment were monitored and chosen through the inclusion (normal health and behavior, and initial weight did not exceed ± 20%), and exclusion criteria (deviation of health, behavior, and weight). The sample size for pharmacokinetics analysis and establishing the most suitable xenograft model was calculated by using the “resource equation” method (E = Total number of animals − Total number of groups). The sample size for the tumor (xenograft) suppression model was found based on the rule of thumb and literature search. The procedures related to the animal’s allocation to a specific experimental group and the injection of substances and cells, and the procedures for measuring the volume of tumors and blood sampling were carried out by different employees who acted independently of each other.

#### Pharmacokinetics and bioavailability of small molecule (MK2.III) using CD-1 mouse line

We investigated the pharmacokinetics (PK) and bioavailability of small molecules (MK2.III) with intraperitoneal and intravenous injection before studying its tumor suppressor properties in combination with DNA-damaging agents. For this set of experiments CD-1 mouse line (male,7 weeks, body weight of 23–25 g, *n* = 198, Charles River) was used with a one-time intraperitoneal injection (IP) of 2, 10, and 50 mg/kg doses of the MK2.III or 2 mg/kg intravenous injection (IV) of the same drug. The small molecule was prepared using PBS containing 10% DMSO. MK2.III was isolated from mouse serum using protein settling method with acetonitrile and drug concentrations were detected using HPLC – MS/MS.

Blood samples were obtained from the retro-orbital sinus (at 5, 10, 20, 30, 60, 120, 240, 480, and 14,140 min for IV groups; at 5, 15, 30,60, 120, 240, 480, and 14,140 min for IP groups, respectively) in polypropylene tubes containing 20 μl of 5% EDTA. Blood plasma was separated by centrifugation at 1500 g for 10 min and was stored at -80 °C until analysis. Data were obtained from six animals per time.

An Agilent 1260 Infinity system, a hybrid triple-quadrupole mass spectrometer, and QTRAP 5500 electrospray ionization were used for determining the MK2.III concentration in blood plasma. Separations were achieved using a YMC Triart C18 50 × 2,1 mm, 1,9 μm column. The mobile phase consisted of A (0.1% aqueous formic acid) and B (0.1% formic acid in acetonitrile). The gradient elution t = 0, A:B 95:5; t = 2 min A:B 5:95; t = 2.1 A:B 5:95; t = 2.2 A:B 95:5; t = 2.8 A:B 95:5; flow rate was 0.5 mL min − 1; Retention time—2.8 min. The injection volume was 2 μL. The temperature of the column was set at 40 °C. Tolbutamide was selected as an internal standard. Thawed plasma (45 μL) was transferred to a microtube (1.1 mL volume), treated with a standard solution (5 μL) of the analytes in acetonitrile: water (1:1), stirred on a vortex mixer for 10 s, treated with tolbutamide solution (200 ng/ml), stirred again on the vortex mixer for 10 s, and then proteins were precipitated in the cold at + 4 °C for 15 min, centrifuged for 10 min at 1500 g. The supernatant (150 µl) was transferred to a clean 96-well plate for HPLC–MS/MS analysis. Samples were stored at a temperature not exceeding -70 °C until analysis. The calculation of pharmacokinetic parameters was performed by using Phoenix® WinNonlin® software, version 6.3 (Pharsight Corp., Cary, NC, USA).

#### A pilot analysis of the most suitable xenograft models with two mouse lines, BALB/c Nude, and SCID

To establish the most suitable xenograft model for the main experiment we used two mouse lines – BALB/c Nude and SCID (both female, 6 weeks, *n* = 20, Charles River). Both mouse lines were injected with NCI-H69 cancer cells (xenograft). Before injection, cells were grown at 37 °C in RPMI-1640 medium containing 10% FBS and pen/strep. Cells were mixed with Matrigel (1:1) and depending on the group 5 × 10^6^ or 10 × 10^6^ cells were injected into a mouse subcutaneously along the spine to the level of the left scapula. Mice were divided into 4 groups: (1) 5 × 10^6 NCI-H69 SCID (*n* = 5); (2) 5 × 10^6 NCI-H69 Nude (*n* = 5); (3) 10 × 10^6 NCI-H69 SCID (*n* = 5); and (4) 10 × 10^6 NCI-H69 Nude (*n* = 5). The combination therapy consisted of etoposide and MK2.III and involved two phases: on the preparatory phase, NCI-H69 cells were grown in vitro until the necessary population of cells (5 × 10^6^) was achieved for injection in the main phase.

Results shown in Supplement Fig. 1 demonstrated that overall NCI-H69 cells formed tumors in both mouse lines which grew exponentially with time. Although both mouse lines have shown progressive tumor growth, high concentrations of injected cells (10 × 10^6^ cells) resulted in accelerated growth 14 days after injection. The stable and progressive growth seen in the SCID mouse line injected with 5 × 10^6^ cells compared to BALB/c Nude mouse using the same concentration of injected cells makes the SCID mouse line more suitable for the animal model and further xenograft experiments.

#### Final xenograft experiments using combination chemotherapy in immunosuppressed SCID mice

The main experiment (injection of the drugs) took place after the tumor growth was established. The tumor growth in mice following the injection of cells was monitored twice a week and upon reaching the volume of ~ 100 mm^3^, animals were randomized into 5 groups (12 female SCID mice in each group). The drug treatment was performed as follows: the first group was a control and received a placebo in the form of PBS (tumor volume 95 ± 38 mm^3^); the second group received 12 mg/kg of Etoposide (tumor volume 101 ± 51 mm^3^); the third group was injected with 4 mg/kg of MK2.III (tumor volume 105 ± 44 mm^3^); fourth and fifth groups were injected with 12 mg/kg of Etoposide and 4 mg/kg or 2 mg/kg of MK2.III (tumor volumes 112 ± 57 and 107 ± 49 mm^3^ respectively). Drugs were injected intraperitoneally daily. Etoposide was injected 3 days after the randomization into groups and MK2.III was administered for 28 days. Tumor growth/suppression was measured using calipers every 3–4 days for the duration of the 28-day treatment and the tumor volume was calculated as follows:

1 $$\mathrm V=\mathrm L\;\mathrm x\;\mathrm W^2/2,\;\mathrm{where}\;\mathrm L\;\mathrm{is}\;\mathrm{length},\;\mathrm W\;\mathrm{is}\;\mathrm{tumor}\;\mathrm{width}\;\mathrm{volume},$$

Antitumor properties of drugs in the test group were determined based on the slowing of the tumor growth or tumor volume compared with the control group receiving PBS. Animal welfare was monitored throughout the study period to ensure that animals in distress were euthanized by carbon dioxide asphyxiation. All the animals were also euthanized by carbon dioxide asphyxiation following the completion of the study.

### Statistical analysis

Descriptive statistics were used to summarize the tumor size and data reported as medians and 25th and 75th quartiles. Wilcoxon sum rank test was used to investigate the effects of the treatments on tumor growth. Values were considered significantly different at the *p* ≤ 0.05 level. Statistical analyses were performed on the SigmaPlot 11.0 software.

## Results

### Cell viability of NCI—H69 cells treated with etoposide, MK2.III and SB203580

Initially, we assessed the response of NCI-H69 SCLC cells to etoposide, p38MAPK inhibitor SB203580, and MK2 inhibitor MK2.III to check on the reproducibility and to establish the optimal concentration of p38MAPK and MK2 inhibitors to use. The concentrations used were 0.312, 0.625, 1.25, 2.5, and 5.0 µM for SB203580 and MK2.III and 10, 20, 50, 100, 150 µM for etoposide. The results are shown in Table 1.

**Table 1:** NCI-H69 cell viability dose response

**Agent**	**Dose in µM**	**Viable cells (%)**
DMSO (0.2%)		88.5
VP-16 (Etoposide)	10	75.7
	20	76.8
	50	65. 2
	100	61.3
	150	55.4
MK2.III	0.321	87.4
	0.626	90.2
	1.25	87.3
	2.5	88.4
	5.0	83.5
SB203580	0.321	89
	0.626	89.8
	1.25	89
	2.5	89.4
	5.0	85.1

Our data demonstrated that control NCI-H69 cells (treated only with the carrier molecule DMSO) showed a good cell viability score of 88.5%. Treatment of NCI-H69 cells using etoposide resulted in decreased cell viability with increasing etoposide dose. These decreases in cell viability are moderate to high; however, this should be a suitable range for the assessment of any synergy between etoposide and kinase inhibitors.

In the presence of MK2.III we observed a cell viability ranging from 87.4% at the lowest dose to 83.5% at the highest dose of MK2.III, suggesting that MK2.III by itself has little effect on NCI-H69 cell viability compared to DMSO treatment alone, except for a small effect at the highest MK2.III dose. Using increasing doses of SB203580 gave a cell viability score ranging from 89% to 85.1% compared to 88.5% in the DMSO control, showing that SB203580 treatment has little effect on the viability of NCI-H69 cells compared to DMSO. Our preliminary experimental data on drug titrations (not shown) allowed us to use 2.5 µM SB203580 and 1.0 µM MK2.III in further experiments.

Thus, NCI-H69 cells were treated with etoposide at doses from 0 µM to 150 µM combined with SB203580 at 2.5 µM, or MK2.III at 1.0 µM respectively and subjected to FACS analysis for viability. The results are presented in Supplement Figs. 2–4 and tabulated in Table 2. Using etoposide alone resulted in cell viability in control samples ranging from 57.3% at the dose of 20 µM to 37.5% at the highest dose Table 2, Supplement Fig. 2). Although the effects of etoposide and p38MAPK/MK2 inhibitors on NCI-H69 cell viability in this set of the experiments are larger than seen in Table 1, they are within the variability seen in repeated experiments (data not shown).

**Table 2:** Effect of kinase inhibitors combined with etoposide on NCI-H69 cell viability

**VP-16 (dose in µM)**	**Viable cells (%)**		
	VP-16	VP-16 + MK2.III (1.0 µM)	VP-16 + SB203580 (2.5 µM)
**0**	74.6	66.1	75.7
**20**	57.3	22.3	45.3
**50**	46.4	24.8	30.9
**100**	45.4	24.1	31.3
**150**	37.5	23	27.4

SB203580 alone at 2.5 µM did not affect NCI-H69 cell viability compared to the DMSO control. However, SB203580 showed a moderate effect on cell viability when used in combination with etoposide (in concentrations ranging from 20 to 150 µM) compared to etoposide alone (Table 2, Supplement Fig. 3). These data suggest a moderate degree of synergy between these agents. In turn, the effect of 1.0 µM MK2.III on NCI-H69 cells was moderate, decreasing cell viability from 74.6% to 66.1% (Table 2, Supplement Fig. 3). However, the combined effects of MK2.III at 1.0 µM and etoposide were large, with the viability decreasing to 22–25% even at low etoposide doses (Table 2, Supplement Fig. 4). This suggests a substantial synergy between these agents.

**Fig. 1:**
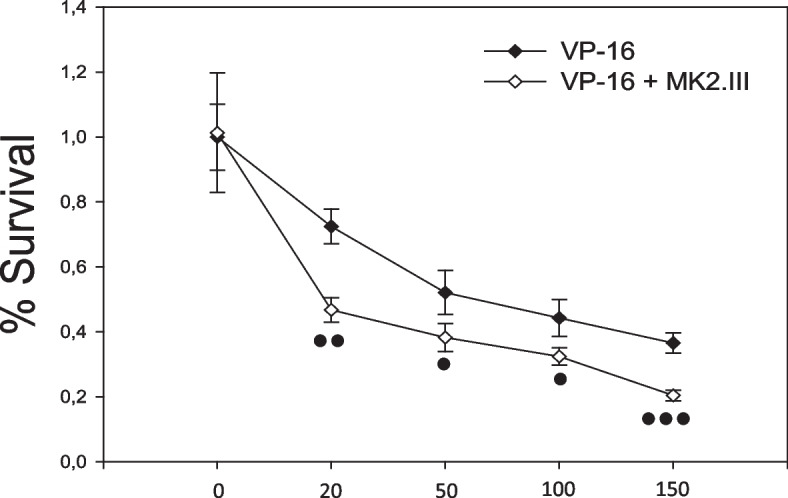
CCK8 cell viability assay of NCI-H69 cells treated with etoposide in concentrations ranging from 0 to 150 μM and 1 μM of MK2.III. ••• - *p* ≤ 0.001, •• - *p* ≤ 0.01, • - *p* ≤ 0.05 compared to the group treated with etoposide only. Data are presented from three independent sets of experiments

In agreement with the FACS-based cell viability assay, CCK8 analysis has revealed similar dynamics demonstrating a significant decrease in the survival rate of NCI-H69 cells treated with etoposide in concentrations ranging from 20 to 150 μM and 1 μM of MK2.III compared to the cells incubated with etoposide alone (Fig. 1). As can be seen from the figure etoposide reduced cell viability in a dose-dependent manner. The percentage of surviving cells varies in a range from 72 to 36% in dosages from 20 μM to 150 μM. Simultaneous treatment of etoposide with MK2.III leads to viability decreasing in all doses when compared to cells treated with Etoposide alone.

### Cell cycle profile of NCI-H69 cells treated with etoposide, MK2.III and SB203580

**Fig. 2:**
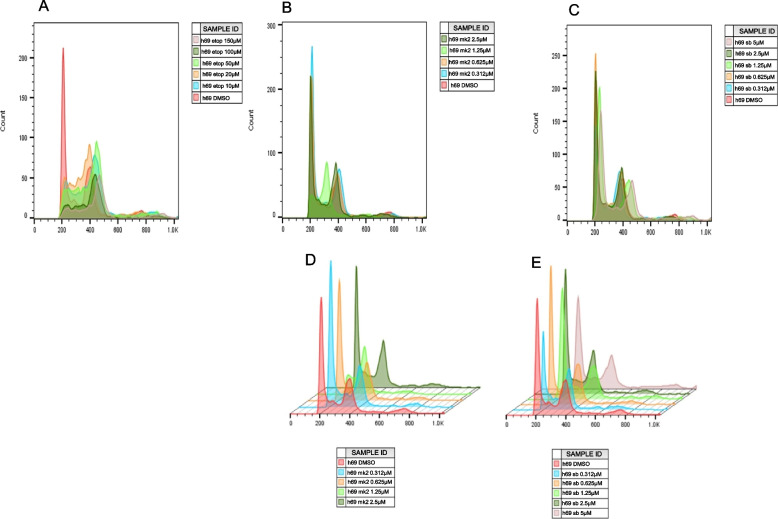
Cell cycle profile of H69 cells treated with etoposide (**A**), MK2.III (**B**), and SB203580 (**C**); (**D**) – 3 dimension of (**B**); (**E**) – 3 dimension of (**C**). Note: the MK2 5 µM sample was lost during the experiment

**Fig. 3:**
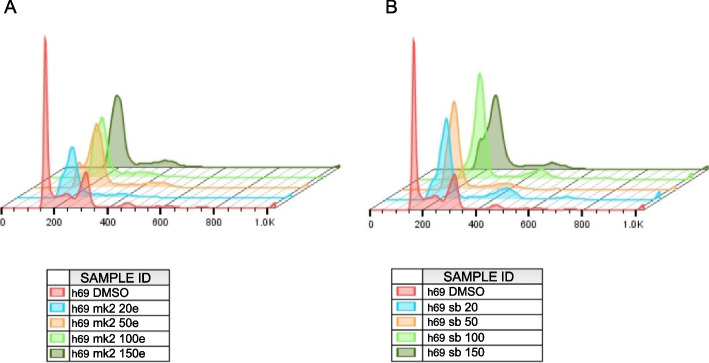
Cell cycle profile of H69 cells treated with etoposide (0, 20, 50, 100, 150 µM) in combination with either MK2.III at 1.0 µM (**A**) or SB203580 at 2.5 µM (**B**)

Treatment of NCI-H69 cells with DMSO results in a normal cell cycle DNA profile with a large G1 and small G2 peaks (Fig. 2A). Treatment with etoposide (Fig. 2A) shows a pronounced reduction in the size of the G1 peak with increasing etoposide dose, indicative of a G2 cell cycle arrest. The G2 peak shifts predominantly to the right with an increasing dose of DNA damaging agent. Treatment of NCI-H69 cells with the MK2.III shows little effect on the cell cycle profile (Fig. 2B, D) showing no obvious G1 or G2 cell arrest. A similar effect on the cell cycle profile was seen in NCI-H69 cells with SB203580 (Fig. 2C, E). At the same time, cell cycle analysis of etoposide-treated cells in combination with either MK2.III (Fig. 3A) or SB203580 (Fig. 3B) show a G1 peak but a very small, if not absent G2 peak, suggestive of a G1 arrest in these cells compared to the single treatment profiles (Fig. 3).

### Pharmacokinetics and bioavailability of small molecule (MK2.III) with intraperitoneal and intravenous injection

To further test our hypothesis, we have assessed the anti-tumor properties of MK2.III targeting the G2 phase of the cell cycle when used in combination with etoposide in immunosuppressed mice. We have chosen to use MK2.III for the in vivo work rather than SB203580 as the former is more effective at reducing cell viability in combination with etoposide based upon the in vitro experiments (see Table 2). The cell line NCL-H69 of human small-cell lung carcinoma was chosen for in vivo experiments because it has a mutation in the P53 gene (p.E171*), a moderate sensitivity to etoposide [28], and is widely used for studies of chemoresistance of cancer [29–31].

We investigated the pharmacokinetics and bioavailability of small molecules (MK2.III) with intraperitoneal and intravenous injection before studying its tumor suppressor properties in combination with DNA-damaging agents. The results of the one-time intraperitoneal injection with three doses of the small molecules shown in Table 3 revealed that 2 mg/kg administration of the drug through intraperitoneal injection showed moderate absorbance of the small molecule in the serum of mice with Tmax of 2 h and Cmax of 2.15 ng/ml while higher concentrations of the drug resulted in a higher yield of the drug within 30 min after injection showing Cmax around 40 ng/ml. T ½ for 2 mg/kg dose was not detected while T ½ for 10 mg/kg and 50 mg/kg doses were 14.4 and 28.3 h respectively. Median serum concentrations and pharmacokinetic curve for MK2.III after 2 mg/kg intravenous injection is demonstrated in Supplement Fig. 5. As can be seen from the figure, the highest concentration of the drug in serum at the time of the injection was 11 ng/ml which decreased up to 2.15 ng/ml in the one-hour interval. Serum concentrations of the drug then gradually decreased within 2 and 4 h respectively. This shows the pharmacokinetics of the small molecule—MK2.III does not represent linearity in the range of 2–50 mg/kg dose. The absolute bioavailability of the MK2.III is illustrated in Supplement Table 1. Due to the nonlinear nature of the MK2.III pharmacokinetics the absolute bioavailability (F_abs_, %), and median absorbance time of the drug reflected in diverse values.

**Table 3:** Pharmacokinetics parameters of MK2.III inhibitor in mice serum with 2, 10 and 50 mg/kg intraperitoneal injection

**Parameters**	**Units**	**Dose**		
		**2 mg/kg, *n* = 48**	**10 mg/kg *n* = 48**	**50 mg/kg, *n* = 48**
Kel	1/hr	n/d*	0,05	0,02
T1/2	hr	n/d	14,4	28,3
Tmax	hr	2	0,5	0,5
Cmax	ng/ml	2,15	38,3	42,4
AUClast	hr*ng/ml	1,99	130	227
AUCinf	hr*ng/ml	n/d	203	548
Vz/F	l/kg	n/d	1027	3718
Cl/F	ml/min/kg	n/d	821	1520
MRTlast	hr	1,28	8,59	10,3
MRTinf	hr	n/d	21,7	42,3

^*^ - non detectable

### Effectiveness of combination therapy on tumor (xenograft) suppression with etoposide and MK2.III in immunosuppressed mice

**Fig. 4:**
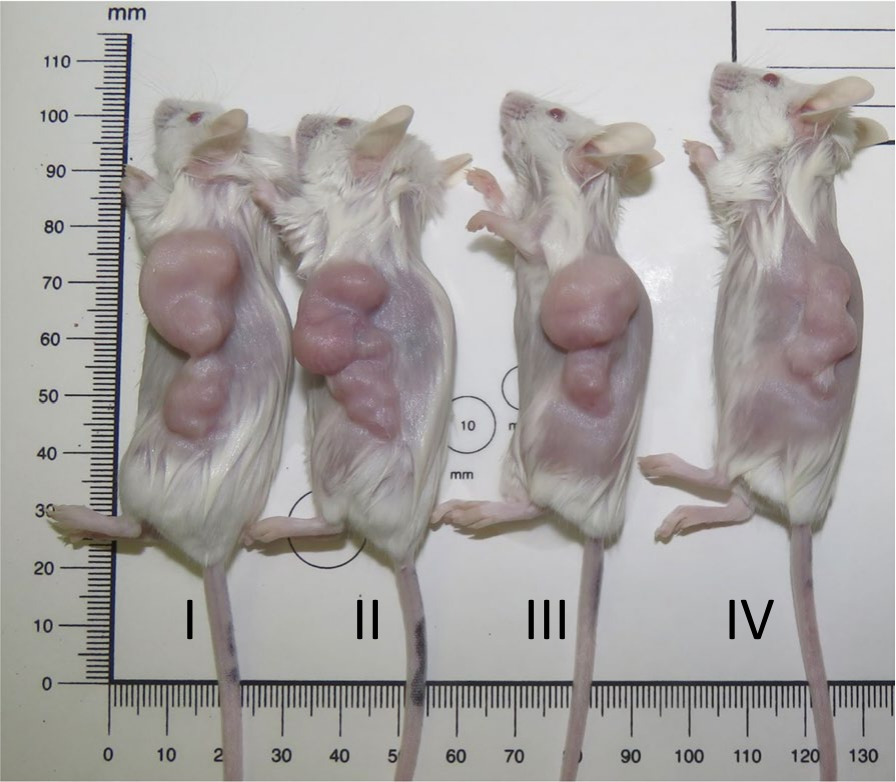
Representative images of tumor size in immunosuppressed SCID mice treated with etoposide and MK2.III on the 18th day after treatment: I—control; II—12 mg/kg of Etoposide; III—4 mg/kg of MK2.III; IV—12 mg/kg of Etoposide and 4 mg/kg of MK2.III

Based on pharmacokinetics, median serum concentrations, and absolute bioavailability of the MK2.III after intraperitoneal injection, as well as previously published data [32], for further in vivo experiments we have chosen 2 mg/kg and 4 mg/kg of MK2.III. The results of the combination therapy performed with etoposide (VP-16) and MK2.III is shown in Table 4. Results of the in vivo study revealed that there was no significant difference between the control, single etoposide, or MK2.III administration or etoposide + MK2.III (2 mg/kg) groups on tumor size at any checkpoint (data not presented). We have also observed no differences in tumor growth dynamics in etoposide + MK2.III group (4 mg/kg) up to seven days (data not presented). However, combination therapy with etoposide + MK2.III (4 mg/kg) resulted in a significant reduction in tumor volume on the 11^th^ and 18^th^ days of the treatment compared to the control group. Moreover, on the 18^th^ day of the experiment, a significant reduction in tumor growth took place in etoposide + MK2.III group compared to single etoposide treatment. Representative images of tumor size in immunosuppressed SCID mice treated with etoposide and MK2.III on the 18th day after treatment is shown in Fig. 4. Although we have not observed any differences in tumor size after the 25^th^ day of the experiment, this finding is still a proof of concept of our initial hypothesis that outlines the role of the MK2 signaling pathway in combination therapy during cancer treatment. Furthermore, this indicates that an inhibitor of the MK2 pathway, small molecule—MK2.III can act as a chemosensitizer when used in combination with DNA-damaging agents, particularly etoposide, making NCI-H69 lung cancer cells sensitive to chemotherapeutic drug for up to ~ 45% compared to single usage of the drug.

**Table 4:** Tumor volume in SCID mouse line

**Days upon treatment**	**Tumor Volume (mm**^**3**^**)** **median (25**^**th**^** and 75**^**th**^** quartile)**			
	Control *n* = 12	VP-16 (12 mg/kg), *n* = 12	MK2.III (4 mg/kg), *n* = 12	VP-16 + MK2.III *n* = 12
**-1**	85 (66.5;113)	93.5 (66;125)	106 (72;132)	103 (76;132)
**11**	677(408;769)^*^	556(360;839)	516 (401;703)	447 (341;584)
**18**	956 (874;1175)^**^	1219 (995;1387)^***^	843 (712;1197)	673 (619;822)

^*^p = 0.05186 (control vs V-16 + MK2.II at day 11)

^**^p = 0.01004 (control vs V-16 + MK2.II at day 18)

^***^p = 0.006812 (V-16 vs V-16 + MK2.II at day 18)

### CCK8 cell viability assay of MDA-MB-231, SW620 and SW480 cancer cell lines

**Fig. 5:**
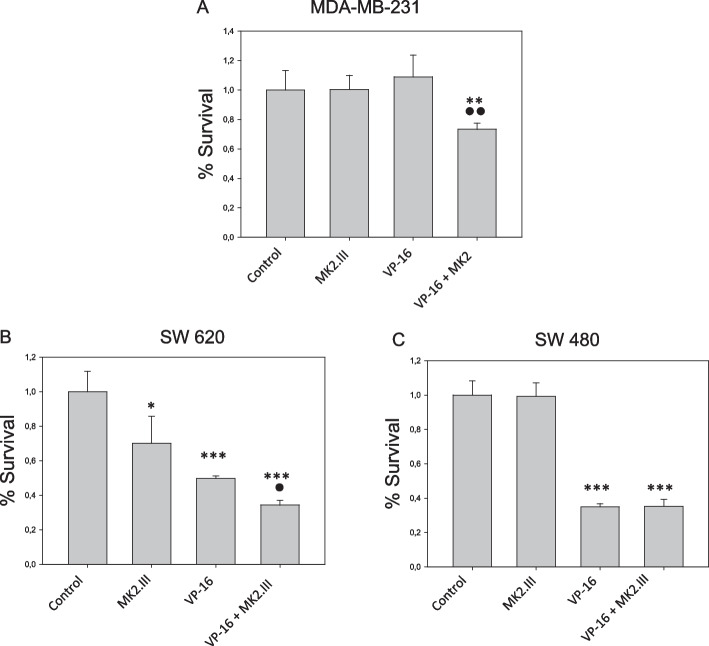
CCK8 cell viability assay of MDA-MB-231 (**A**), SW620 (**B**), and SW480 (**C**) cancer cell lines treated with 150 μM Etoposide and 1 μM of MK2.III. *** - *p* ≤ 0.001, ** - *p* ≤ 0.01, * - *p* ≤ 0.05 compared to the control; •• - *p* ≤ 0.01, • - *p* ≤ 0.05 compared to the group treated only with etoposide. Data are presented from three independent sets of experiments

To further confirm the synergy effects of etoposide and MK2.III on p53 mutant cancer cells, additional CCK8 cell viability analysis of MDA-MB-231 breast cancer cell line, and SW620, and SW480 colorectal cancer cell lines was performed (Fig. 5). The assay has shown that MK2.III alone does not affect the viability of MDA-MB-231 and SW480 cells while decreasing the viability of SW620 by 30%. The viability of the SW620 and SW480 cells incubated with etoposide alone decreased by 50% and 65% respectively, and yet, etoposide alone did not affect the viability of MDA-MB-231 cells. However, MDA-MB-231 as well as SW620 cells treated with etoposide and MK2.III showed diminished viability compared to the cells that were incubated with sole etoposide (by 27% and 31%, respectively). Nonetheless, MK2.III. did not increase sensitivity to etoposide of SW480 cells.

## Discussion

In the current study, we have investigated the ability of p38MAPK/MK2 inhibitors to enhance the sensitivity of cancer cells lacking G1 checkpoint (NCI-H69 lung cancer cells) to DNA-damaging anticancer drug etoposide. Results of the co-treatment experiments have demonstrated that the p38MAPK/MK2 kinase inhibitors SB203580 and MK2.III both enhanced the DNA-damaging effects of etoposide on NCI-H69 cell viability in vitro. In vivo, results revealed that MK2.III was able to act as a chemosensitizer when used in combination with etoposide making NCI-H69 lung cancer cells more sensitive to the chemotherapeutic drug. We have also analyzed the cell cycle profile of NCI-H69 cells treated with etoposide, MK2.III and SB203580. Our data suggest SCLC cells arrest in the G1 phase of the cell cycle compared to the single treatment profiles.

Some cancer cells have abrogated G1 checkpoints depending on their p53 status, the tumor suppressor molecule involved in the regulation of cellular senescence possibly as a response mechanism to DNA damage [10]. However, those cancer cells still have activated G2 checkpoint, a chromatin-quality checkpoint in late G2 involving ATR/p38MAPK/MK2 [14–16, 24]. Genetic disruption of the p38MAPK/MK2 pathway can specifically sensitize p53-null mouse cells to DNA-damaging agents [27, 33]. The cancer cells that lack p53 with abrogated p38MAPK/MK2 pathways are unable to go through G1 and G2 DNA damage checkpoints and will enter mitosis with damaged DNA where they undergo apoptosis by “mitotic catastrophe”. Nevertheless, p53 wild-type cells are still able to arrest in response to DNA damage as the p53-dependent G1 checkpoint in those cells remain intact making such cells halt in G1 and avoid mitotic catastrophe. This is a novel approach to therapy in cancer treatment as the inhibitors of p38MAPK and MK2 inhibitors selectively target cells depending on their p53-null status thus leaving healthy cells intact.

Several p38MAPK and MK2 pathway inhibitors have previously shown the ability to enhance the effectiveness of chemotherapeutic agents when used in combination. Some studies have shown that SB203580 inhibited the activity of p38MAPK in gastric cancer cells (BGC823) consequently improving the sensitivity of BGC823 cells to doxorubicin and induced cell death [34]. Furthermore, co-treatment with SB202190 and irinotecan improved the sensitivity of chemoresistant colorectal cancer cells to chemotherapy [35], and a p38αMAPK-selective inhibitor SCIO-469 reduced tumor growth in multiple myeloma xenograft tumors by enhancing the effect of bortezomib [23]. An inhibitor of p38MAPK BIRB796 inhibited proliferation and invasion in U87 and U251glioblastoma cells in vitro [36] and enhanced the antitumor effects of chemotherapeutic agents in cervical cancer [37] and human oral epidermoid carcinoma cell line [38] both vitro and in vivo. However, several attempts to use the p38MAPK pathway as a therapeutic target for cancer treatment failed due to side effects on the heart, liver, and nervous system associated with p38MAPK inhibitor toxicities [39].

Compared to p38MAPK the MK2 pathway has been less studied, however, some data suggest that MK2 knockdown reduces in vivo growth of multiple myeloma in mouse models with MK2 overexpression leading to bortezomib and doxorubicin chemoresistance by reducing apoptosis [40]. In addition, a combination of MK2 inhibitor IV with bortezomib, doxorubicin, or dexamethasone suppressed the proliferation of multiple myeloma cells and improved survival in mouse models [41]. In 311our study, we have utilized the small molecule MK2 inhibitor III (MK2.III). MK2.III belongs to a relatively novel class of MK2 inhibitors with potent cellular activity based on the pyrrolo-pyrimidone core structure [42, 43]. MK2.III is an ATP-competitive inhibitor of MK2 with a high degree of selectivity [42, 43], however, limited information is available regarding its chemo-sensitizing effects. Besides, the pharmacokinetics of MK2.III has not been previously reported yet. Therefore, we have investigated the combined effects of etoposide and MK2.III on p53 mutated cancer cells in vitro and in vivo as well as pharmacokinetics and bioavailability of MK2.III injected intraperitoneally and intravenously.

It has been reported that the effect of MK2.III on the sensitivity of cancer cells to chemotherapy depends on the chemotherapy drug. For example, inhibition of MK2 by MK2.III was shown to sensitize p53 mutated pancreatic cancer cells (Panc1 and MIA PaCa-2) and osteosarcoma (U2OS) to cisplatin [44], and, in contrast, protect cancer cells from gemcitabine [44–46]. In our study, we utilized the SCLC cell line NCI-H69. According to published data, small cell lung cancer (SCLC) is the most malignant among the various types of lung cancer, characterized by the emergence of drug resistance, which is still a serious clinical problem [29]. Standard first-line therapy for metastatic SCLC involves treatment with the topoisomerase II inhibitor II etoposide [47], however, many cases of etoposide-resistant lung cancer have been identified in recent years [28]. We have chosen the NCL-H69 cell line of human small-cell lung carcinoma due to its mutated status in the P53 gene (p.E171*) and moderate sensitivity to etoposide [28]. In our study, the combined effects of MK2.III at 1.0 µM and etoposide are large compared to SB203580, with the viability decreasing to 22–25% even at low etoposide doses.

We have also performed additional experiments using colorectal cancer cell lines SW480 and SW620, and breast cancer cell line MDA-MB-231. MDA-MB-231 is a human breast cancer cell line derived from the metastatic breast adenocarcinoma of a 51-year-old Caucasian woman. The cells of this line are p53 mutant, metastatic, and very invasive [48, 49]. SW-480 and SW-620 colorectal cancer cells were isolated from primary colon adenocarcinoma (SW-480) and lymph node metastasis (SW-620) of the same patient [50, 51]. However, despite the similar origin and p53-null status, the cells of these two lines respond distinctly to cellular stress: SW480 line demonstrates a normal DNA repair phenotype [51]. In our experiments, we observed that MK2.III sensitized metastatic cell lines SW-620 and MDA-MB-231 to etoposide, whereas, MK2.III exposure did not increase the sensitivity of non-metastasizing SW-480 colorectal cells to anti-cancer drug.

In vivo*,* combination chemotherapy of etoposide and MK2.III at a dose of 4 mg/kg on tumor xenografts in immunosuppressed mice resulted in up to a 45% decrease in tumor growth compared to single usage of the drug after 18 days of treatment. Although we have not observed any differences in tumor sizes among treatment groups starting from the 25^th^ day of the experiment, this might be attributed to the limitations of our research design, since only one treatment regimen (3 daily injections of etoposide and 28 daily injections of MK2.III) and only two MK2.III doses were tested. Furthermore, during treatment mice may increase their ability to excrete MK2.III with time. This may indicate that MK2.III is effective at the early stages of treatment once reaches serum, however, naturally gets reduced due to decreased serum levels, and repeated dosing is needed. In fact, MK2.III is an ATP competitive inhibitor and an increased level of intracellular ATP may also reduce its effectiveness [52]. One possible factor contributing to the increased ATP levels could be the rise in extracellular ATP (eATP) concentration triggered by cell death [53]. Consequently, cancer cells have the capability to internalize eATP, leading to a significant increase in intracellular ATP levels, which, in turn, promotes cell proliferation and drug resistance [54]. Therefore, the concentration of MK2.III must be controlled due to non-linear pharmacokinetics and its possible competition with ATP and more studies are needed to address this issue.

As mentioned, the pharmacokinetics of MK2.III have not been previously reported and only limited information is available regarding pharmacological profile of MK2.III analogs [43, 55]. As an example, nonlinear pharmacokinetics have been described following oral administration of the pyrrolo-pyrimidones inhibitor MK2 (Compound 1) in rats [55]. In our study, we have found nonlinear PH of MK2.III as well. Besides, we have shown that the pharmacokinetic profile of MK2.III with peritoneal administration was improved compared to oral administration. We suggest that the nonlinearity of the pharmacokinetics of MK2.III is attributed to its physicochemical properties, in particular, the poor solubility of the inhibitor [52]. In addition, one of the reasons for non-linear FC may be the slow penetration of the drug through the serous surfaces with the administration of elevated concentrations of the inhibitor. On the other hand, despite poor solubility and low bioavailability, pyrrolo-pyrimidones inhibitors are weakly bound to blood and culture medium proteins, which in turn can allow high blood (or plasma) concentrations of the free drug to be achieved [43].

## Conclusions

In conclusion, our data suggest that inhibiting the activity of MK2 signaling pathways in the G2 phase of the cell cycle using small molecules are attractive targets for further research in chemoresistance. This approach may not be unique to SCLC cells but can also be explored in other cell lines of various cancers. Hence, it may open future perspectives on studying the role of the MK2 pathway and its inhibitors in combination therapy during cancer treatment. This data is undoubtedly a key finding in improving the effectiveness of the chemotherapy, which uses the strategy to lower the doses of the DNA damaging agents with the help of small molecules (inhibitors of the MK2 pathway). Findings reported in this study provide evidence that specific inhibitors of MK2 may indeed improve overall cancer therapy; however, their effectiveness depends on cell types. As cancer treatment moves towards a more personalized medical approach, proper diagnosis paired with targeted and informed approaches to treating specific types of cancer with in-depth knowledge of chemoresistance may prove to be a useful strategy for overcoming drug treatment failures that ultimately lead to recurrence and death.

### Supplementary Information

**Additional file 1: Supplement Figure 1.** Tumor growth in BALB/c Nude and SCID immunosuppressed mouse lines (*n*= 48). Data is presented as M +/- SEM. **Supplement Figure 2.** FACS analysis of the dose response on H69 cells with Etoposide (data in Table 2). Cell viability is demonstrated by Draq7 positive staining. **Supplement Figure 3.** FACS analysis of the dose response on H69 cells with Etoposide in the presence of SB203580 at 2.5 µM (data in Table 2). Cell viability is demonstrated by Draq7 positive staining. **Supplement Figure 4.** FACS analysis of the dose response on H69 cells with Etoposide in the presence of MK2.III at 1.0µM (data in Table 2). Cell viability is demonstrated by Draq7 positive staining. **Supplement Figure 5.** Average serum concentrations of MK2.III inhibitor in mice with 2 mg/kg intravenous administration. **Supplement Table 1.** Absolute bioavailability (F_abs_, %) and median absorbance time (МАТ) of MK2.III inhibitor in mice serum with 2 , 10 and 50 mg/kg intraperitoneal injection.

## Data Availability

Data available on request. To access data Dauren Alimbetov and Sholpan Akarova should be contacted (alimbetov@uthscsa.edu, shaskarova@nu.edu.kz, Tel.: + 7 7172 70 65 14).

## References

[1] Dominguez-Brauer C (2015). Targeting mitosis in cancer: emerging strategies. Mol Cell.

[2] Mirzayans R (2012). New insights into p53 signaling and cancer cell response to DNA damage: implications for cancer therapy. J Biomed Biotechnol.

[3] Zhao H, Piwnica-Worms H (2001). ATR-mediated checkpoint pathways regulate phosphorylation and activation of human Chk1. Mol Cell Biol.

[4] Zhao H, Watkins JL, Piwnica-Worms H (2002). Disruption of the checkpoint kinase 1/cell division cycle 25A pathway abrogates ionizing radiation-induced S and G2 checkpoints. Proc Natl Acad Sci U S A.

[5] Matthews HK, Bertoli C, de Bruin RAM (2022). Cell cycle control in cancer. Nat Rev Mol Cell Biol.

[6] Alimbetov D, et al. Pharmacological targeting of cell cycle, apoptotic and cell adhesion signaling pathways implicated in chemoresistance of cancer cells. Int J Mol Sci. 2018;19. 10.3390/ijms19061690. 10.3390/ijms19061690 PMC6032165 29882812

[7] Woo MG (2012). Calpain-mediated processing of p53-associated parkin-like cytoplasmic protein (PARC) affects chemosensitivity of human ovarian cancer cells by promoting p53 subcellular trafficking. J Biol Chem.

[8] Muller PA, Vousden KH (2013). p53 mutations in cancer. Nat Cell Biol.

[9] Xue W (2007). Senescence and tumour clearance is triggered by p53 restoration in murine liver carcinomas. Nature.

[10] Lehmann BD, Pietenpol JA (2012). Targeting mutant p53 in human tumors. J Clin Oncol.

[11] Fan S (1995). Disruption of p53 function sensitizes breast cancer MCF-7 cells to cisplatin and pentoxifylline. Cancer Res.

[12] Ma CX, Janetka JW, Piwnica-Worms H (2011). Death by releasing the breaks: CHK1 inhibitors as cancer therapeutics. Trends Mol Med.

[13] Visconti R, Della Monica R, Grieco D (2016). Cell cycle checkpoint in cancer: a therapeutically targetable double-edged sword. J Exp Clin Cancer Res.

[14] Reinhardt HC (2007). p53-deficient cells rely on ATM- and ATR-mediated checkpoint signaling through the p38MAPK/MK2 pathway for survival after DNA damage. Cancer Cell.

[15] Mikhailov A, Shinohara M, Rieder CL (2004). Topoisomerase II and histone deacetylase inhibitors delay the G2/M transition by triggering the p38 MAPK checkpoint pathway. J Cell Biol.

[16] Mikhailov A, Shinohara M, Rieder CL (2005). The p38-mediated stress-activated checkpoint. A rapid response system for delaying progression through antephase and entry into mitosis. Cell Cycle.

[17] Sanchez-Prieto R (2000). A role for the p38 mitogen-acitvated protein kinase pathway in the transcriptional activation of p53 on genotoxic stress by chemotherapeutic agents. Cancer Res.

[18] Cai B (2006). p38 MAP kinase mediates apoptosis through phosphorylation of BimEL at Ser-65. J Biol Chem.

[19] Clark A, Dean J (2012). The p38 MAPK pathway in rheumatoid arthritis: a sideways look. Open Rheumatol J.

[20] Correa S, Eales K (2012). The role of p38 MAPK and its substrates in neuronal plasticity and neurodegenerative disease. J Signal Transduction.

[21] Wagner E, Nebreda A, Wagner EF, Nebreda AR (2009). Signal integration by JNK and p38 MAPK pathways in cancer development. Nat Rev Cancer.

[22] Martínez-Limón A (2020). The p38 pathway: from biology to cancer therapy. Int J Mol Sci.

[23] Grossi V (2014). p38α MAPK pathway: a key factor in colorectal cancer therapy and chemoresistance. World J Gastroenterol.

[24] Manke IA (2005). MAPKAP kinase-2 is a cell cycle checkpoint kinase that regulates the G2/M transition and S phase progression in response to UV irradiation. Mol Cell.

[25] Pan ST (2016). Molecular mechanisms for tumour resistance to chemotherapy. Clin Exp Pharmacol Physiol.

[26] Igea A, Nebreda AR (2015). The stress kinase p38α as a target for cancer therapy. Cancer Res.

[27] Bucher N, Britten CD (2008). G2 checkpoint abrogation and checkpoint kinase-1 targeting in the treatment of cancer. Br J Cancer.

[28] Qiu Z (2019). A novel mutation panel for predicting etoposide resistance in small-cell lung cancer. Drug Des Devel Ther.

[29] Gardner EE (2017). Chemosensitive relapse in small cell lung cancer proceeds through an EZH2-SLFN11 Axis. Cancer Cell.

[30] Campling BG (1991). Chemosensitivity testing of small cell lung cancer using the MTT assay. Br J Cancer.

[31] Cañadas I (2014). Targeting epithelial-to-mesenchymal transition with Met inhibitors reverts chemoresistance in small cell lung cancer. Clin Cancer Res.

[32] Liu X, Wu T, Chi P (2013). Inhibition of MK2 shows promise for preventing postoperative ileus in mice. J Surg Res.

[33] Morandell S (2013). A reversible gene-targeting strategy identifies synthetic lethal interactions between MK2 and p53 in the DNA damage response in vivo. Cell Rep.

[34] Tan W, Yu HG, Luo HS (2014). Inhibition of the p38 MAPK pathway sensitizes human gastric cells to doxorubicin treatment in vitro and in vivo. Mol Med Rep.

[35] Paillas S (2011). Targeting the p38 MAPK pathway inhibits irinotecan resistance in colon adenocarcinoma. Cancer Res.

[36] Zhao L (2021). BIRB796, an Inhibitor of p38 Mitogen-Activated Protein Kinase, Inhibits Proliferation and Invasion in Glioblastoma Cells. ACS Omega.

[37] Jin X (2016). The p38 MAPK inhibitor BIRB796 enhances the antitumor effects of VX680 in cervical cancer. Cancer Biol Ther.

[38] He D (2013). BIRB796, the inhibitor of p38 mitogen-activated protein kinase, enhances the efficacy of chemotherapeutic agents in ABCB1 overexpression cells. PLoS One.

[39] Soni S, Anand P, Padwad Y (2019). MAPKAPK2: The master regulator of RNA-binding proteins modulates transcript stability and tumor progression. J Exp Clin Cancer Res.

[40] Gu C (2021). MK2 is a therapeutic target for high-risk multiple myeloma. Haematologica.

[41] Guo M (2019). Targeting MK2 is a novel approach to interfere in multiple myeloma. Front Oncol.

[42] Schlapbach A (2008). Pyrrolo-pyrimidones: a novel class of MK2 inhibitors with potent cellular activity. Bioorg Med Chem Lett.

[43] Anderson DR (2007). Pyrrolopyridine Inhibitors of Mitogen-Activated Protein Kinase-Activated Protein Kinase 2 (MK-2). J Med Chem.

[44] Li Y, Köpper F, Dobbelstein M (2018). Inhibition of MAPKAPK2/MK2 facilitates DNA replication upon cancer cell treatment with gemcitabine but not cisplatin. Cancer Lett.

[45] Köpper F (2013). Damage-induced DNA replication stalling relies on MAPK-activated protein kinase 2 activity. Proc Natl Acad Sci.

[46] Köpper F (2014). The MAPK-activated protein kinase 2 mediates gemcitabine sensitivity in pancreatic cancer cells. Cell Cycle.

[47] Kalemkerian GP (2013). Small cell lung cancer. J Natl Compr Canc Netw.

[48] Olivier M (2002). The IARC TP53 database: new online mutation analysis and recommendations to users. Hum Mutat.

[49] Hui L (2006). Mutant p53 in MDA-MB-231 breast cancer cells is stabilized by elevated phospholipase D activity and contributes to survival signals generated by phospholipase D. Oncogene.

[50] Rochette PJ (2005). SW480, a p53 double-mutant cell line retains proficiency for some p53 functions. J Mol Biol.

[51] Lamy V (2010). p53 activates either survival or apoptotic signaling responses in lupulone-treated human colon adenocarcinoma cells and derived metastatic cells. Transl Oncol.

[52] Fiore M, Forli S, Manetti F (2016). Targeting Mitogen-Activated Protein Kinase-Activated Protein Kinase 2 (MAPKAPK2, MK2): Medicinal Chemistry Efforts To Lead Small Molecule Inhibitors to Clinical Trials. J Med Chem.

[53] Antonioli L (2013). Immunity, inflammation and cancer: a leading role for adenosine. Nat Rev Cancer.

[54] Qian Y (2016). Extracellular ATP a New Player in Cancer Metabolism: NSCLC Cells Internalize ATP In Vitro and In Vivo Using Multiple Endocytic Mechanisms. Mol Cancer Res.

[55] Chiang P-C (2009). Aqueous versus non-aqueous salt delivery strategies to enhance oral bioavailability of a mitogen-activated protein kinase-activated protein kinase (MK-2) inhibitor in rats. J Pharm Sci.

