# Supplementary material for: Small molecule targeting of the p38/Mk2 stress signaling pathways to improve cancer treatment
Source: BMC Cancer. 2023 Sep 23;23:895. doi: 10.1186/s12885-023-11319-x (PMC10517462; doi:10.1186/s12885-023-11319-x)
Supplement: Supplementary file 1 — Additional file 1: Supplement Figure 1. Tumor growth in BALB/c Nude and SCID immunosuppressed mouse lines (n= 48). Data is presented as M +/- SEM. Supplement Figure 2. FACS analysis of the dose response on H69 cells with Etoposide (data in Table 2). Cell viability is demonstrated by Draq7 positive staining. Supplement Figure 3. FACS analysis of the dose response on H69 cells with Etoposide in the presence of SB203580 at 2.5 µM (data in Table 2). Cell viability is demonstrated by Draq7 positive staining. Supplement Figure 4. FACS analysis of the dose response on H69 cells with Etoposide in the presence of MK2.III at 1.0µM (data in Table 2). Cell viability is demonstrated by Draq7 positive staining. Supplement Figure 5. Average serum concentrations of MK2.III inhibitor in mice with 2 mg/kg intravenous administration. Supplement Table 1. Absolute bioavailability (Fabs, %) and median absorbance time (МАТ) of MK2.III inhibitor in mice serum with 2 , 10 and 50 mg/kg intraperitoneal injection. [file 12885_2023_11319_MOESM1_ESM.pptx]

## Slide 1
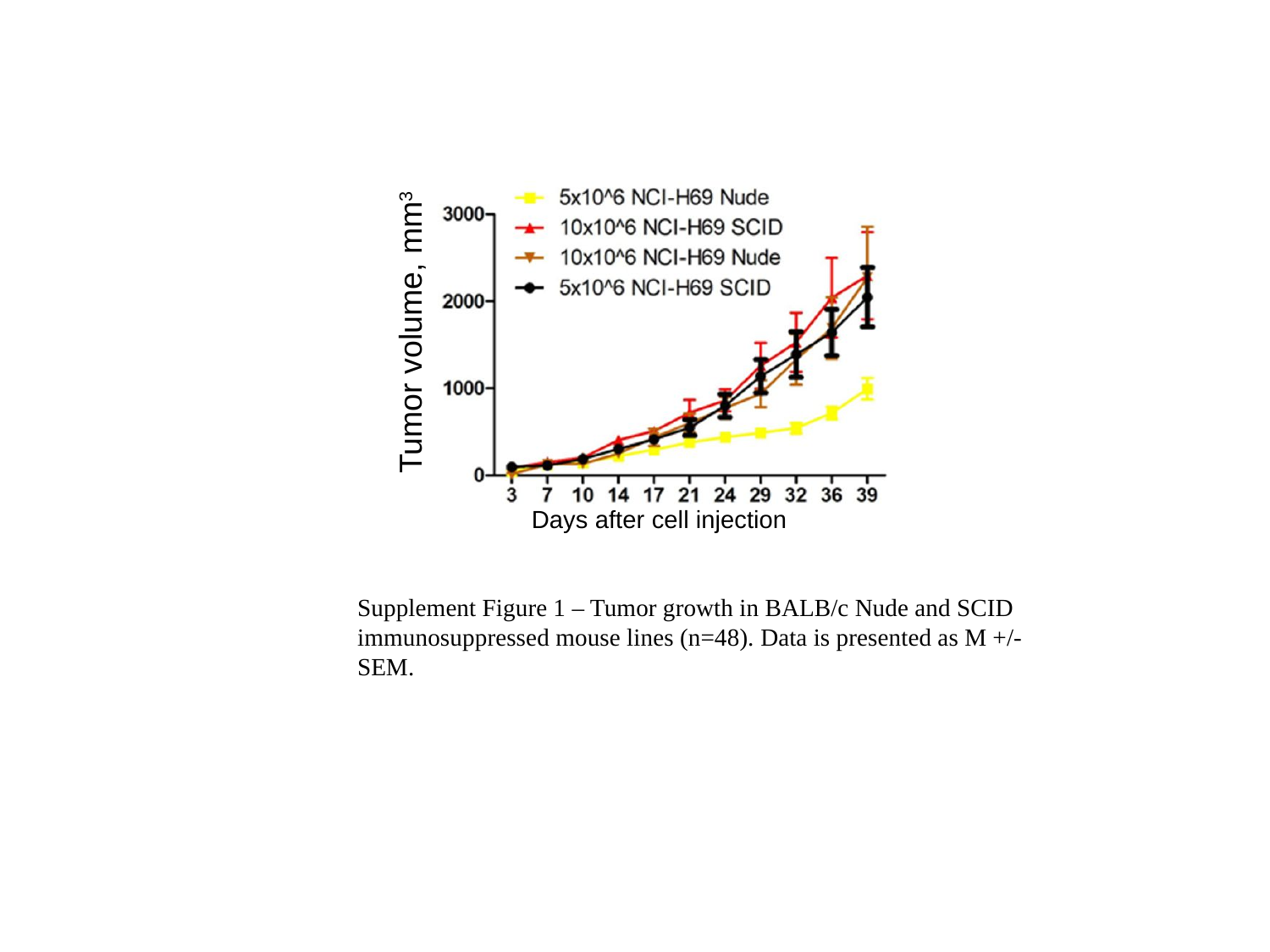

Tumor volume, mm3
Days after cell injection
Supplement Figure 1 – Tumor growth in BALB/c Nude and SCID immunosuppressed mouse lines (n=48). Data is presented as M +/- SEM.

## Slide 2
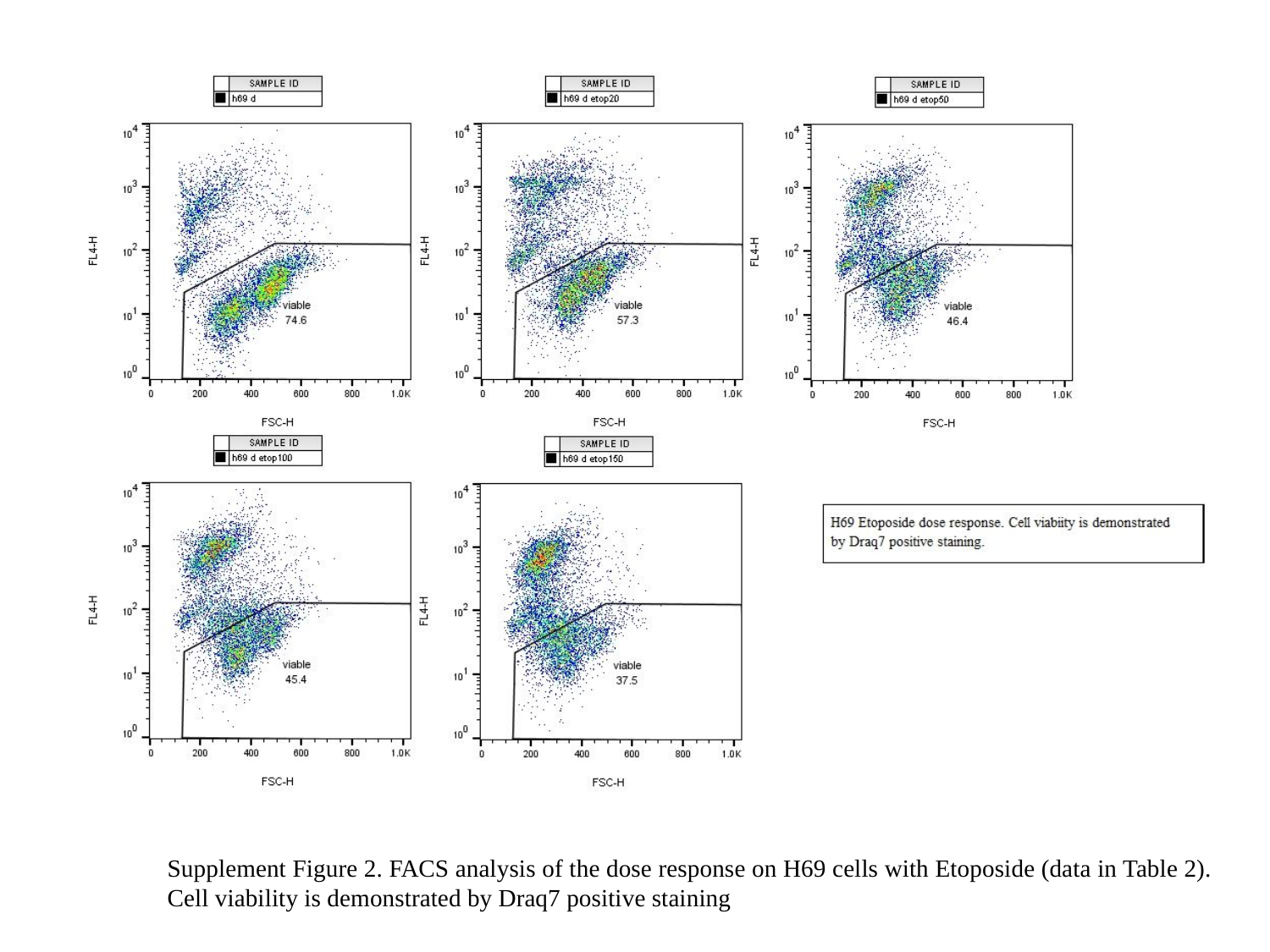

Supplement Figure 2. FACS analysis of the dose response on H69 cells with Etoposide (data in Table 2). Cell viability is demonstrated by Draq7 positive staining

## Slide 3
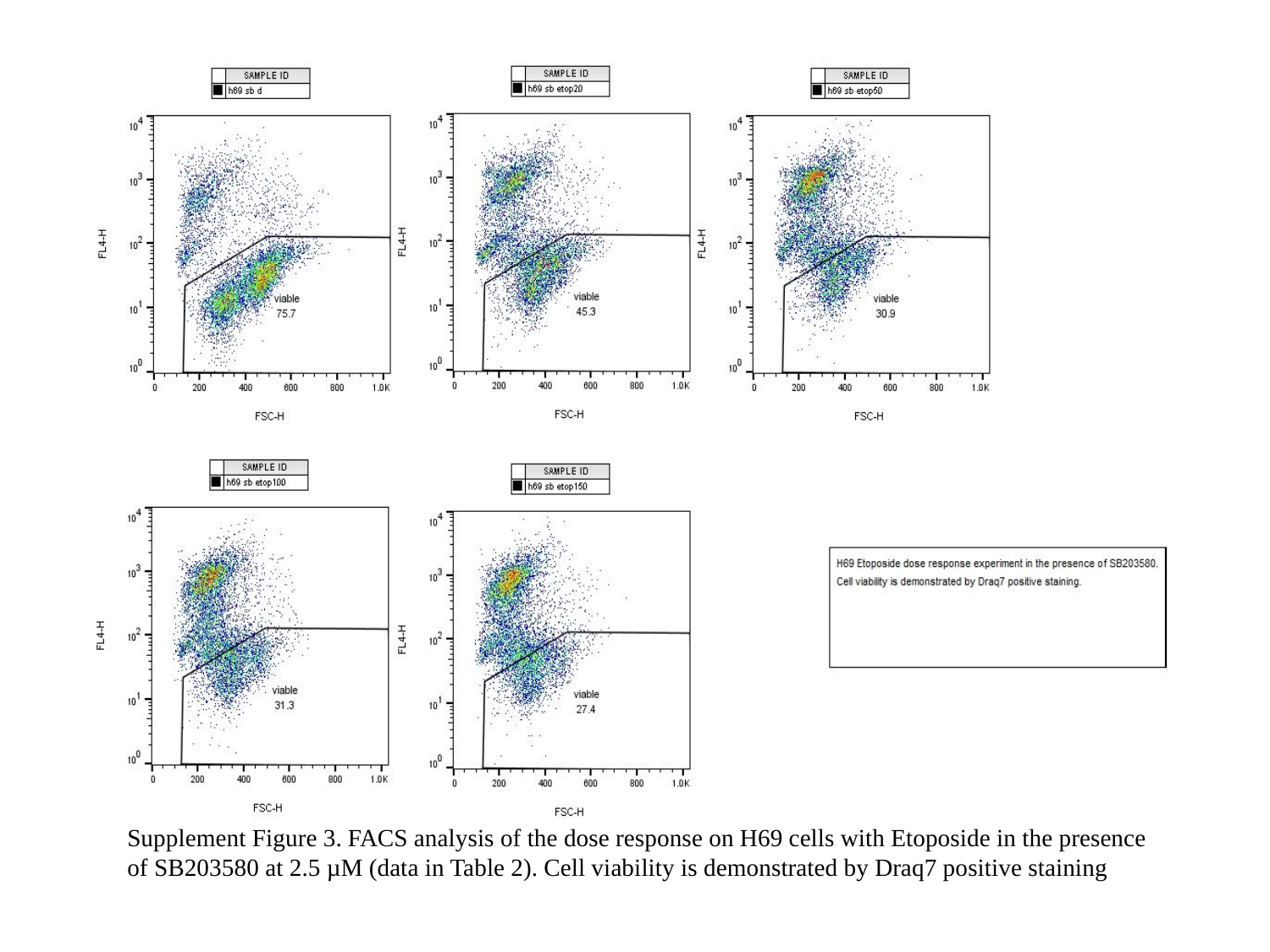

Supplement Figure 3. FACS analysis of the dose response on H69 cells with Etoposide in the presence of SB203580 at 2.5 µM (data in Table 2). Cell viability is demonstrated by Draq7 positive staining

## Slide 4
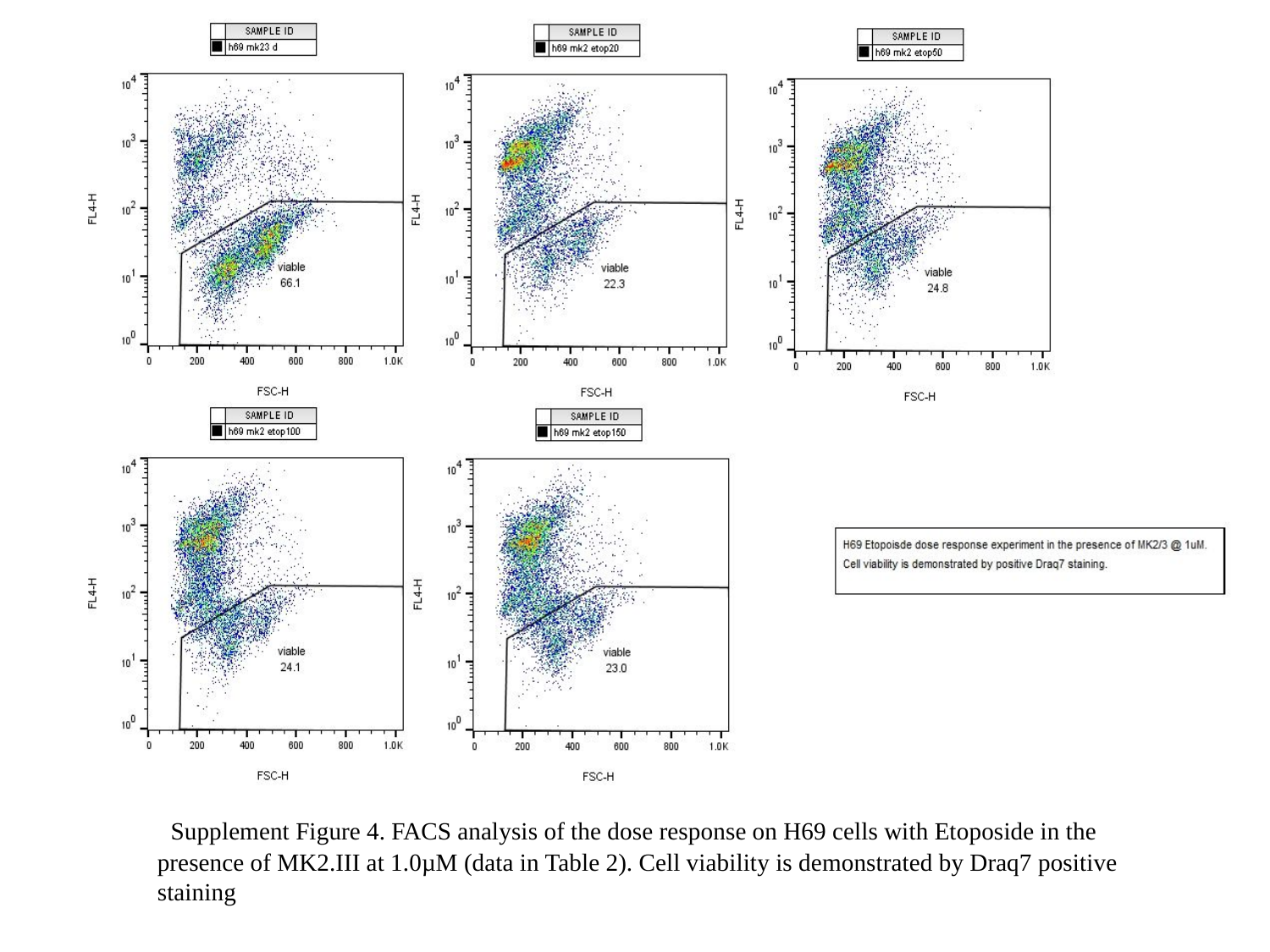

Supplement Figure 4. FACS analysis of the dose response on H69 cells with Etoposide in the presence of MK2.III at 1.0µM (data in Table 2). Cell viability is demonstrated by Draq7 positive staining

## Slide 5
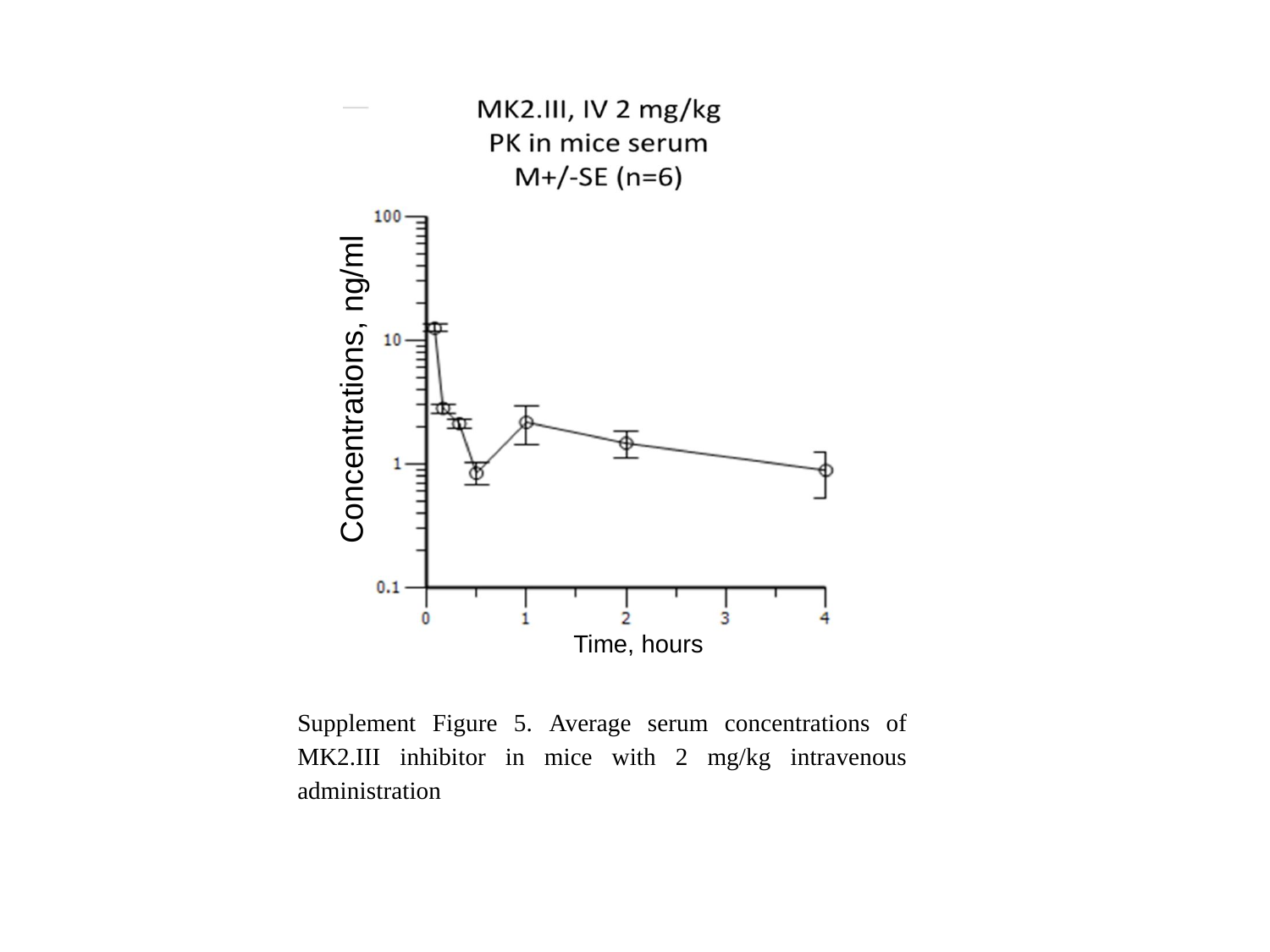

Concentrations, ng/ml
Time, hours
Supplement Figure 5. Average serum concentrations of MK2.III inhibitor in mice with 2 mg/kg intravenous administration

## Slide 6
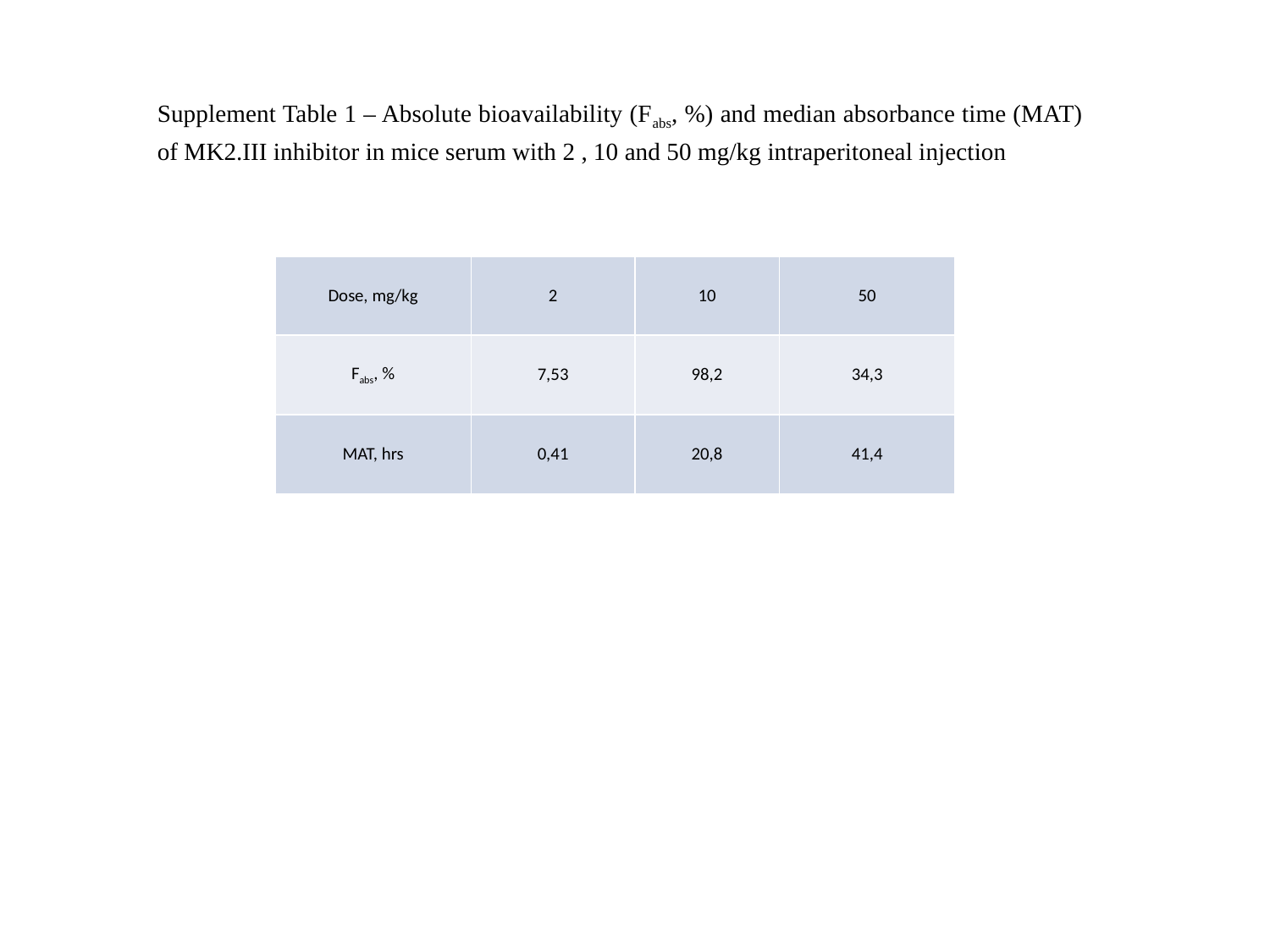

Supplement Table 1 – Absolute bioavailability (Fabs, %) and median absorbance time (МАТ) of MK2.III inhibitor in mice serum with 2 , 10 and 50 mg/kg intraperitoneal injection
| Dose, mg/kg | 2 | 10 | 50 |
| --- | --- | --- | --- |
| Fabs, % | 7,53 | 98,2 | 34,3 |
| MAT, hrs | 0,41 | 20,8 | 41,4 |
